# Factors Associated with HIV Testing among Male Students Who Have Engaged in Sexual Behaviour in Zhejiang Province, China

**DOI:** 10.1155/2023/6646210

**Published:** 2023-11-13

**Authors:** Zhongrong Yang, Hui Wang, Qiaoqin Ma, Weiyong Chen, Xiang Zhao, Tingting Jiang, Wanjun Chen, Xin Zhou, Lin Chen

**Affiliations:** ^1^Huzhou Center for Disease Control and Prevention, Huzhou 313000, Zhejiang Province, China; ^2^Zhejiang Provincial Center for Disease Control and Prevention, Hangzhou 310051, Zhejiang Province, China

## Abstract

**Objective:**

This study aimed to estimate the prevalence of human immunodeficiency virus (HIV) testing, identify factors associated with HIV testing among male students who have engaged in sexual behaviour in Zhejiang province, and provide a scientific basis for the prevention and control of HIV infection on campus.

**Methods:**

Stratified cluster random sampling analysis was performed, which included general characteristics, sexual attitudes, sexual behaviours, information on HIV testing, and self-risk assessment for HIV infection. Univariate and multivariate logistic regression analyses were conducted to identify the influencing factors.

**Results:**

Among 2734 male students who have engaged in sexual behaviour, 319 (11.7%) had undergone HIV antibody testing in the previous year. The results of multivariate analysis demonstrated that the participants who were in the junior grade level (adjusted odds ratio (AOR) = 1.59, 95% confidence interval (95% CI): 1.10–2.30) exhibited acceptance to male homosexual behaviour (AOR = 1.73, 95% CI: 1.19–2.52), had been exposed to testing publicity in the previous year (AOR = 1.51, 95% CI: 1.06–2.15), had been exposed to self-risk assessment for HIV infection (AOR = 2.66, 95% CI: 1.99–3.55), had male or bisexual partners (AOR = 1.60, 95% CI: 1.05–2.46), had a score for the scale indicating awareness of different testing methods between 2 and 5 (AOR = 2.19, 95% CI: 1.51–3.16) or greater than 6 (AOR = 1.49, 95% CI: 1.01–2.66), and had a score for the scale indicating knowledge of different testing facilities between 3 and 5 (AOR = 1.63, 95% CI: 1.00–2.66) were inclined to engage in HIV testing.

**Conclusions:**

In this study, the proportion of HIV-testing among male students who have engaged in sexual behaviour was low. This study revealed that students who exhibited acceptance to male homosexual behaviours had been exposed to publicity for HIV testing or a self-risk assessment for HIV infection which were more inclined to engage in HIV testing. Our study underscores the urgent need to enhance educational interventions concerning HIV risks and warnings as part of the health education curriculum on campus. The graveness of the AIDS epidemic among students necessitates this emphasis. Moreover, we recommend deploying condom-dispensing machines or HIV testing facilities across the campus for easy access to preventive and testing services for HIV.

## 1. Introduction

Currently, the epidemics of AIDS and HIV infection remain major public health problems worldwide [1–3]. HIV-infected patients require lifelong medication, and currently no vaccine is available for preventing HIV infection [4]. In recent years, the number of young HIV-infected students in China has increased [5, 6], and sexual transmission (especially male homosexual behaviour) has emerged as the main route of HIV transmission among young students [7, 8]. In Chinese universities, 24.2% of students below 18 years of age actively engage in sexual activities [9]. Meanwhile, 4% of male university students in China engage in sexual behaviours with men [10]. The situation of the AIDS epidemic amongst students in Zhejiang province remains daunting in recent years, predominantly among male students and specifically among men who have sex with men in this demographic [11]. It is notable that the sexual partners of these students often engage socially with other segments of the population. Concurrently, the rate of HIV detection among students is distressingly low, thereby delaying timely identification of those living with HIV [12].

University students who attended movies or parties, were older, engaged in low physical activity, and exhibited liberal sexual attitudes were more inclined to engage in risky sexual behaviours [13, 14]. Furthermore, many college students lack professional sexual education and are unaware of the correct condoms usage and prevention methods for HIV and sexually transmitted diseases (STDs), which could make them more vulnerable to HIV infection [15].

Young students, being in a sexually active phase of life, exhibit open sexual attitudes and are easily influenced by social culture and driven by curiosity, which leads to risky sexual behaviours [16]. Moreover, young students have weak ability to identify AIDS risks and lack the AIDS risk-coping ability, making them easily prone to HIV infection [9]. HIV testing is the only way to confirm that an individual is infected with HIV, and HIV testing/counseling improves the ability of HIV-negative individuals to adopt protective measures and can help HIV-positive people to adopt healthy sexual practices [17].

The promotion of HIV testing is also an important component of the interventions for HIV prevention. Many people living with HIV are unaware of their infection status [18]. The Joint United Nations Programme on HIV (UNAIDS) proposes that HIV testing should be used as an important strategy to reduce HIV infection among individuals aged 15–24 years. HIV testing is critical for achieving UNAIDS 95-95-95 goals and ending the AIDS epidemic [19, 20].

The need for thorough and directed research on factors affecting HIV testing, particularly among male students who have engaged in sexual behaviour, is both substantial and time-sensitive. This urgency is rooted in the pivotal role HIV testing assumes in managing and curbing the AIDS epidemic as the forefront of prevention, care, and treatment modalities. Timely testing and knowledge of one's HIV status can facilitate immediate initiation of antiretroviral therapy for positive individuals, thereby enhancing their life quality and mitigating further transmission. The insights gained through this comprehensive research could potentially sway policy-making and health interventions, having a profound impact on public health.

The HIV testing rate among young Chinese students is low [12], and the reasons for and factors influencing HIV testing remain unclear. To address this knowledge gap, this study estimated the prevalence of HIV testing and identified the factors associated with HIV testing among male students who have engaged in sexual behaviour. The study findings provided a scientific basis for formulating HIV testing and behaviour-based interventions for college students.

## 2. Materials and Methods

### 2.1. Study Participants

In this study, a cross-sectional survey of 13 colleges/universities in 11 districts and cities in Zhejiang province, China, was conducted, from October 2018 to November 2018 [21, 22]. Our study focused on universities or vocational technical colleges, as recommended by the local Center for Disease Control and Prevention [21]. This study adopted a stratified cluster sampling method, whereby three departments were sampled from each school using the random number table method. Each department was divided into four layers based on grades 1–4, and classes were selected from each layer using the random number table method, resulting in a total of 1241 classes [23]. Within each department and grade, 200 students were randomly selected, leading to a total of 800 people selected from grades one to four. In each selected department, classes were numbered according to grades, and then, classes were selected. All students from the selected classes were invited to participate in the survey. If the number of students from a particular class that participated in the survey was fewer than 200, another class was randomly selected. A total of 31674 students participated in the baseline survey, including 14,320 male and 17354 female students. Among these, 2734 male students who have engaged in sexual behaviour were included in the analysis.

### 2.2. Questionnaire Development

Based on a review of domestic and foreign literature and repeated discussions and modifications, a questionnaire was developed by the research team and referred to the AIDS/STDs surveillance questionnaire for students from national center for AIDS/STDs control and prevention of China CDC [24], then a pilot survey was conducted among a class of students in a college. The main contents of the final questionnaire included the demographic characteristics, HIV-relevant knowledge, exposure to different educational levels, sexual attitudes, sexual behaviours, and knowledge and experience of HIV testing services of the participants.

### 2.3. Ethical Consideration

The participants were informed that the study was designed to develop strategies for preventing HIV and STDs among students. They were assured that the survey was anonymous, their privacy would be strictly protected, and the participants had the freedom to decide whether or not to participate in the survey. The survey only analyzed the aggregate data of the group and did not analyze the individual data of the participants. This study was conducted in accordance with the guidelines of the Declaration of Helsinki and approved by the Ethics Committee of the Zhejiang Provincial Center for Disease Control and Prevention (batch number: 2018-036). All participants signed informed consent forms [22]. All methods were performed in accordance with the relevant guidelines and regulations.

### 2.4. Measures

The participants self-reported whether they had undergone HIV testing in the previous year, which was used as the dependent or outcome variable in this study. Demographics, sexual attitudes, exposure to publicity on health education and HIV testing, self-risk assessment for HIV infection, sexual behaviours, scales measuring awareness of different testing methods, and scales measuring knowledge of different testing facilities were evaluated as independent variables. Self-risk assessment for HIV infection included the presence or absence of multiple sexual partners, adherence to condom use, presence or absence of same-sex male partners, presence or absence of temporary/commercial sexual partners, presence or absence of previous diagnosis of sexually transmitted diseases, and whether alcohol or stimulants were used before sexual activity.

The scale used to measure participants' score of having heard of different testing methods included six statements on the following items: voluntary counseling and testing, HIV self-testing, online appointment HIV testing, blood rapid testing, saliva rapid testing, and urine testing. Possible responses to these six statements were “yes” and “no,” which were assigned scores of 1 and 0, respectively. The scale scores range from 0 to 6, where a score of 6 indicates a high level, scores of 2 to 5 indicate a middle level, and scores of 0 to 1 indicate a low level of knowledge regarding HIV testing methods. The Cronbach's alpha coefficient for this scale was 0.902.

The scale for measuring participants' knowledge of different testing facilities included five facility types: the Center for Disease Control and Prevention, general hospitals, community health service centers, college hospitals or clinics, and community-based organizations. Possible responses to these five statements were “yes” and “no,” which were assigned scores of 1 and 0, respectively. The scores on this scale range from 0 to 5, with scores of 3–5 reflecting a high level, 2 reflecting a middle level, and 0–1 reflecting a low level of knowledge about HIV testing providers. The Cronbach's alpha coefficient for this scale was 0.770.

### 2.5. Data Analysis

Data analysis was performed using the SPSS software (version 21.0; SPSS Inc., Chicago, IL, USA). The *χ*^2^ test was performed to compare the differences of receiving HIV testing during the previous year between male students who have engaged in sexual behaviour and those who did not. The association between the dependent variable and each independent variable was first computed using a univariate logistic regression analysis and was represented by an odds ratio (OR) with a corresponding 95% confidence interval (95% CI) and a *P* value based on the *χ*^2^ test of proportions. The variables that demonstrated significant association with self-reported HIV testing in the univariate analyses were then included in a multivariate logistic regression model to determine the independent contribution of each factor in predicting self-reported HIV testing. This model was performed using a backward stepwise logistic regression analysis, with a removal criterion of *P* > 0.1. In this study, we conducted the Cronbach's alpha coefficient of “the scale used to measure participants' score of having heard of different testing methods” and “the scale for measuring participants' knowledge of different testing facilities,” and we used the reliability analysis tool in SPSS software for reliability analysis. A significance level of *P* < 0.05 was used to determine statistically significant differences in this study [21].

## 3. Results

### 3.1. General Characteristics

A total of 2734 male students who have engaged in sexual behaviour, of whom 319 (11.7%) underwent HIV testing in the previous year. The mean age of the participants was 20.20 ± 1.41 years (the minimal and maximal ages were 16 years and 28 years, respectively). As presented in Table 1, significant differences were observed between the two groups in terms of age, college attributes, and grades (*P* < 0.05); however, no significant differences were observed in hometown or monthly living expenses between the two groups (*P* > 0.05).

### 3.2. Correlation Analysis of HIV Testing among Participants

In the univariate analysis (Table 2), participants were more likely to undergo HIV testing if they belonged to the age groups 20–21 years (crude odds ratio(COR) = 1.35) or ≥22 years (COR = 1.51), attended a college with municipal attributes (COR = 1.33), were in the sophomore (COR = 1.55) or junior (COR = 1.69) grades, exhibited acceptance to male homosexual behaviours (COR = 2.44), had been exposed to health education on HIV in the previous year (COR = 2.48), had been exposed to publicity for HIV testing in the previous year (COR = 3.02), had been exposed to a self-risk assessment for HIV infection (COR = 3.87), had access to HIV self-testing reagents for sale or collection at school (COR = 2.45), had male or bisexual partners (COR = 1.68), had a casual partner in the previous year (COR = 1.74), had ever had a commercial partner (COR = 2.12), had a score for the scale of indicating awareness of different testing methods between 2 and 5(COR = 2.76) or greater than 6(COR = 3.45), and had a score for the scale of indicating knowledge about different testing facilities between 3 and 5(COR = 2.76).

In the multivariate analysis (Table 3) using the backward stepwise logistic regression model, participants were more likely to undergo HIV testing if they were in junior grade (adjusted odds ratio (AOR) = 1.59; 95% confidence interval (CI): 1.10–2.30), exhibited acceptance to male homosexual behaviours (AOR = 1.73, 95% CI: 1.19–2.52), had been exposed to publicity for HIV testing in the previous year (AOR = 1.51, 95% CI: 1.06–2.15), had been exposed to a self-risk assessment for HIV infection (AOR = 2.66, 95% CI: 1.99–3.55), had male or bisexual sex partners (AOR = 1.60, 95% CI: 1.05–2.46), had a score for the scale of indicating awareness of different testing methods between 2 and 5(AOR = 2.19, 95% CI: 1.51–3.16) or greater than 6 (AOR = 1.49, 95% CI: 1.01–2.66), and had a score for the scale of indicating knowledge about different testing facilities between 3 and 5(AOR = 1.63, 95% CI: 1.00–2.66).

## 4. Discussion

Owing to their sexual activity and weak awareness of protection, young students are prone to unsafe sex, which increases the risk of HIV transmission [12]. In this study, 2734 male students who have engaged in sexual behaviour, the HIV testing rate for this group was 11.7%, and the HIV detection rate was relatively low. HIV testing is a critical step in the implementation of self-protection for young students and plays an important role in helping individuals to understand their infection status, seek antiretroviral therapy, and stop HIV transmission [25].

The results of this study indicated that junior grade students were more inclined to undergo HIV testing than freshman students. As students progressed to higher grades, their exposure to testing publicity increased, along with their knowledge of HIV prevention and treatment, contributing to an increased proportion of HIV testing [26]. Our results also indicated a higher frequency of HIV testing among sophomore and junior students than among senior students. However, no statistical difference was observed among senior students, which could be possibly due to the senior participants occupied with job hunting or graduation thesis preparation at this stage; therefore, they may have neglected to undergo HIV testing.

The results of this study indicated that the participants' HIV testing rate increased by 73% and 60% for those who exhibited acceptance to male homosexual behaviour and had male or bisexual partners, respectively. As this group realized that such sexual behaviours may be risk factors for HIV infection, they were more willing to take the initiative to undergo HIV testing. With an increase in the publicity and promotion of knowledge on HIV prevention and control in recent years by various departments and multiple internet platforms such as WeChat public accounts, forums, and websites, the level of knowledge on HIV health among participants has improved [27]. In the future, we need to emphasize the relationship between unsafe sex and HIV infection in the process of developing students' awareness about AIDS [28]. Meanwhile, we need to improve the awareness of the risk of HIV infection among college students who engage in sexual behaviours, emphasize the necessity of timely detection of HIV after unsafe sexual behaviours, and reduce the occurrence of unsafe sexual behaviours.

A lack of awareness regarding the risk of HIV infection is the most important barrier to HIV testing [29]. Individuals are more likely to take corresponding preventive and protective measures after perceiving their susceptibility. If an individual has good awareness of HIV infection and, simultaneously, the public information on HIV infection closely relates to the individuals' sexual behaviour, the provided information will increase the individual's HIV infection [30]. In this study, 37.3% of the college students were exposed to a self-risk assessment for HIV infection, whereas most college students were not.

Wilbourn et al. demonstrated that adolescents and young adults with a perceived risk of HIV infection were more likely to undergo testing for HIV infection [31]. Our results also revealed that the number of participants who had ever undergone a self-risk assessment for HIV infection was 166% higher among those who underwent testing for HIV infection than among those who did not. Therefore, we need to develop scientific HIV risk assessment standards or systems by establishing the concepts of high-risk behaviours, risk acceptance, goal setting, risk identification, risk analysis, and risk response to HIV infection, which can provide a basis and reference for HIV prevention and control in young students. In addition, we need to develop an HIV risk assessment application program software designed for young students and promote its usage through internet platforms, schoolteachers, and parents. The promotion of behaviour-based HIV testing interventions should focus on improving the perception of HIV susceptibility among students on campus. Furthermore, it is necessary to strengthen HIV infection warning education among college students so that they can recognize the current situation of HIV infection, improve their awareness of HIV infection risk, correctly assess their own HIV infection risk, and effectively reduce risky behaviours.

In this study, participants who were aware of more than two different testing methods were more inclined to engage in HIV testing than those who were aware of two or fewer testing methods. These methods include voluntary HIV counseling and testing [32], HIV self-testing [33], HIV network appointment testing, HIV blood (fingertip/venous blood) rapid testing [34], HIV oral saliva rapid testing [35], and HIV urine testing [36]. At present, many universities in Zhejiang province promote HIV self-testing to encourage young students who engage in high-risk sexual behaviours to undergo HIV testing in a timely manner. It is necessary to strengthen young students' HIV testing-related knowledge in the future, improve the accessibility and convenience of testing locations and times inside and outside schools, and create a safe and convenient testing environment.

In this study, the number of participants who underwent HIV testing was 63% higher among those who knew about more than two testing facilities than among those who knew about two or fewer testing facilities. To increase the utilization of HIV testing, we need to add more HIV testing equipment in or around schools, strengthen public information about testing services and the benefits of early detection of HIV infection, and increase the publicity of AIDS destigmatization through the sharing of peer HIV testing experiences.

In this study, no statistically significant differences were observed in the variables between commercial and casual sexual partners. However, young students who exhibited these behaviours should be tested for HIV infection as soon as possible [37, 38]. HIV testing is the only reliable method to determine whether a sexual partner is infected with HIV, and students who were acquainted with someone living with HIV exhibited increased inclined to undergo HIV testing [39].

This study had several limitations. First, the questionnaires were self-reported, and the survey in this study involved sensitive privacy issues such as sexual behaviour, which may have a certain degree of recall or response bias. We conducted a pilot survey among a class of students in a college, but did not evaluate the questionnaire, which may have an impact on the quality of the survey questionnaire. In addition, owing to the cross-sectional nature of the survey used in this study, the factors that were identified to impact HIV testing may be limited. However, the exploration of sexual behaviour among college students whether undergo HIV testing has a certain level of representativeness, and further investigations needs to be performed in the future to determine the generalizability of our findings. Future studies should focus on investigating the reasons behind the reluctance of the college students who engage in sexual behaviours to undergo HIV testing.

## 5. Conclusions

In this study, the proportion of HIV-testing among male students who have engaged in sexual behaviour was low. This study revealed that students who exhibited acceptance to male homosexual behaviours had been exposed to publicity for HIV testing or a self-risk assessment for HIV infection were more inclined to engage in HIV testing. Our study underscores the urgent need to enhance educational interventions concerning HIV risks and warnings as part of the health education curriculum on campus. The graveness of the AIDS epidemic among students necessitates this emphasis. Moreover, we recommend deploying condom-dispensing machines or HIV testing facilities across the campus for easy access to preventive and testing services for HIV. This research provides policymakers with key insights that can enhance HIV prevention and testing services among male students who have engaged in sexual behaviour. By promoting HIV awareness, encouraging HIV self-testing and risk assessment, and improving access to preventive measures like condoms and testing services on campus, policymakers can help reduce HIV infection rates.

## Figures and Tables

**Table 1:** Demographic characteristics of participants.

Variables	HIV-untested group (*n* = 2415)		HIV-tested group (*n* = 319)		*x* ^2^	*P*
	*n*	%	*n*	%		
*Age group (years)*						
≤19	749	31.0	78	24.5	6.310	0.043
20–21	1266	52.4	178	55.8		
≥22	400	16.6	63	19.7		

*College attributes*						
Provincial	652	27.0	73	22.9	6.262	0.044
Municipal	1383	57.3	206	64.6		
Private	380	15.7	40	12.5		

*Grades*						
Freshman	479	19.8	47	14.7	19.739	<0.001
Sophomore	731	30.3	111	34.8		
Junior	755	31.3	125	39.2		
Senior	450	18.6	36	11.3		

*Hometown*						
City^#^	601	24.9	83	26.0	0.435	0.805
Town^#^	352	14.6	49	15.4		
Rural area	1462	60.5	187	58.6		

*Monthly living expenses (CNY* ^∗^)						
≤1200	767	31.8	114	35.7	2.062	0.357
1201–1500	789	32.7	97	30.4		
>1500	859	35.6	108	33.9		

^*∗*^CNY, Chinese Yuan. ^#^“City” refers to urban area, which generally refers to the location of county-level or above governments in a relatively large area; “Town” usually refers to the location of a township or street government, with a relatively small area.

**Table 2:** Univariate analysis of HIV testing among participants.

Variables	Total *n* (col %)	Tested (*n* (row %)	Crude OR (95% CI)^a^	*P*
*Age group (years)*				
≤19	827 (31.0)	78 (9.4)	Ref	
20–21	1444 (52.4)	178 (12.3)	1.35 (1.02–1.79)	0.036
≥22	463 (16.6)	63 (13.6)	1.51 (1.06–2.15)	0.022

*College attributes*				
Provincial	725 (27.0)	73 (10.1)	Ref	
Municipal	1589 (57.3)	206 (13.0)	1.33 (1.003–1.77)	0.048
Private	420 (15.7)	40 (9.5)	0.94 (0.63–1.41)	0.766

*Grades*				
Freshman	526 (19.8)	47 (8.9)	Ref	
Sophomore	842 (30.3)	111 (13.2)	1.55 (1.08–2.22)	0.017
Junior	880 (31.3)	125 (14.2)	1.69 (1.18–2.41)	0.004
Senior	486 (18.6)	36 (7.4)	0.82 (0.52–1.28)	0.377

*Had accepted one-night stand*				
None/unknown	1327 (48.5)	156 (11.8)	Ref	
Yes	1407 (51.5)	163 (11.6)	0.98 (0.78–1.24)	0.889

*Had accepted commercial sex*				
None/unknown	1859 (68.0)	216 (11.6)	Ref	
Yes	875 (32.0)	103 (11.8)	1.02 (0.79–1.30)	0.908

*Had accepted male homosexual behaviour*				
None/unknown	2414 (88.3)	249 (10.3)	Ref	
Yes	320 (11.7)	70 (21.9)	2.44 (1.81–3.27)	<0.001

*Ever had been exposed to health education on HIV in the previous year*				
No	891 (32.6)	56 (6.3)	Ref	
Yes	1843 (67.4)	263 (14.3)	2.48 (1.84–3.35)	<0.001

*Ever had been exposed to publicity for HIV testing in the previous year*				
No	1040 (38.0)	59 (5.7)	Ref	
Yes	1694 (62.0)	260 (15.3)	3.02 (2.25–4.05)	<0.001

*Ever had been exposed to self-risk assessment for HIV infection*				
No	1713 (62.7)	108 (6.3)	Ref	
Yes	1021 (37.3)	211 (20.7)	3.87 (3.03–4.96)	<0.001

*Whether schools having HIV self-testing reagents for sale or collection*				
No/unknown	2220 (81.2)	213 (9.6)	Ref	
Yes	514 (18.8)	106 (20.6)	2.45 (1.90–3.16)	<0.001

*Gender of sexual partner*				
Female	2446 (89.5)	256 (10.5)	Ref	
Male or bisexual	219 (8.0)	54 (24.7)	1.68 (1.33–2.11)	<0.001

*Had casual sexual partner in the previous year*				
No	2139 (78.2)	220 (10.3)	Ref	
Yes	595 (21.8)	99 (16.6)	1.74 (1.35–2.25)	<0.001

*Ever had commercial sexual partner*				
No	2556 (93.5)	282 (11.0)	Ref	
Yes	178 (6.5)	37 (20.8)	2.12 (1.44–3.10)	<0.001

*Score for the scale of having heard of different testing methods (scores)*				
0–1	917 (33.5)	49 (5.3)	Ref	
2–5	921 (33.7)	124 (13.5)	2.76 (1.95–3.89)	<0.001
6	896 (32.8)	146 (16.3)	3.45 (2.46–4.84)	<0.001

*Score for the scale of having known a different testing facility (scores)*				
0–1	362 (13.2)	24 (6.6)	Ref	
2	1214 (44.4)	105 (8.6)	1.33 (0.84–2.11)	0.220
3–5	1158 (42.4)	190 (16.4)	2.76 (1.78–4.30)	<0.001

^a^OR, odds ratio; CI, confidence interval. ^b^The percentage may not add up to 100% due to missing data.

**Table 3:** Multivariate analysis of HIV testing among participants.

Variables	Adjusted OR (95% CI)^a^	*P*
*Grades*		
Freshman	Ref	
Sophomore	1.36 (0.94–1.98)	0.108
Junior	1.59 (1.10–2.30)	0.014
Senior	0.95 (0.58–1.54)	0.824

*Had accepted male homosexual behaviour*		
None/unknown	Ref	
Yes	1.73 (1.19–2.52)	0.005

*Ever had been exposed to publicity for HIV testing in the previous year*		
No	Ref	
Yes	1.51 (1.06–2.15)	0.021

*Ever had been exposed to self-risk assessment for HIV infection*		
No	Ref	
Yes	2.66 (1.99–3.55)	<0.001

*Gender of sexual partner*		
Female	Ref	
Male or bisexual	1.60 (1.05–2.46)	0.030

*Score for the scale of having heard of different testing methods (scores)*		
0–1	Ref	
2–5	2.19 (1.51–3.16)	<0.001
6	1.49 (1.01–2.66)	0.045

*Score for the scale of having known a different testing facility (scores)*		
0–1	Ref	
2	1.15 (0.70–1.89)	0.57
3–5	1.63 (1.00–2.66)	0.049

^a^OR, odds ratio; CI, confidence interval.

## Data Availability

The datasets generated and analyzed during the current study are available from the corresponding authors upon reasonable request.
